# Frailty predicts short-term incidence of future falls among British community-dwelling older people: a prospective cohort study nested within a randomised controlled trial

**DOI:** 10.1186/s12877-015-0152-7

**Published:** 2015-12-02

**Authors:** Gotaro Kojima, Denise Kendrick, Dawn A. Skelton, Richard W. Morris, Sheena Gawler, Steve Iliffe

**Affiliations:** Department of Primary Care and Population Health, University College London (Royal Free Campus), Rowland Hill Street, London, NW3 2PF UK; School of Medicine, Division of Primary Care, University of Nottingham, Nottingham, UK; School of Health and Life Sciences, Institute of Applied Health Research, Glasgow Caledonian University, Glasgow, UK; School of Social and Community Medicine, University of Bristol, Bristol, UK

**Keywords:** Frailty, Falls, Older people

## Abstract

**Background:**

Although population-based studies have shown frailty predicted future falls, their follow-up periods were one year or longer and short-term fall risks associated with frailty are unknown.

**Methods:**

A prospective cohort study nested within a randomised controlled trial was conducted to examine associations between frailty and short-term incident future falls among community-dwelling older people. Two hundred forty eight community-dwelling people > =65 years without history of > =three falls and allocated to a usual care arm of exercise intervention trial were prospectively monitored for falls over 24 weeks. Frailty index (FI) was constructed from 40 deficits at baseline. The future fall risks according to frailty status was examined using logistic regression models.

**Results:**

Of 248 participants, 46 were classified as frail and 57 had one or more falls during follow-up. Both each 0.01 increase in FI and frailty defined as FI > =0.25 were significantly associated with higher risks of future falls in multivariate logistic regression models adjusted for age, gender and history of two falls in the previous year (odds ratio (OR) = 1.05, 95 % confidence interval (95 % CI) = 1.02–1.07, *p* < 0.001; OR = 3.04, 95 % CI = 1.53–6.02, *p* = 0.001, respectively). Receiver operating characteristic (ROC) curve analysis showed FI predicted future falls with fair accuracy with area under ROC curve of 0.62 (95 % CI = 0.53–0.71, *p* < 0.01).

**Conclusions:**

Frailty was a significant and independent predictor of short-term future falls among community-dwelling older people who had volunteered for a physical activity study. It is important for healthcare practitioners to recognise frailty as a risk factor of imminent future falling even in older people who appear to be ageing well.

## Background

Frailty is a syndrome resulting from age-related cumulative declines across multiple physiological systems characterised by decreased homeostatic reserves and increased vulnerability to adverse health outcomes including disabilities, hospitalisation, institutionalisation, or death [1, 2]. Recent guidelines from the British Geriatric Society advocate assessment of frailty during all encounters with health and social care professionals [2].

Frailty is also recognised as a risk factor for falls [1, 3]. Main features of frailty include weakness, as well as balance and gait problems, all of which predispose older people to falling [1]. Falls in older people often occur as a result of diminished functional reserve capacity involved in maintaining the upright position and vulnerability to internal and external stressors, such as environmental hazards, impairments, disease processes, or adverse pharmacological effects [4]. Approximately 30 % of older people aged 65 years or more and 50 % of those over 80 years fall every year [5]. Not only are falls related to injuries or fractures and are a leading cause of morbidity and mortality in older people, [6] but falls are also shown to have a negative psychological impact. The negative consequences related to falls include fear of falling, loss of confidence, anxiety, depressive symptoms and decreased self-efficacy, which may lead to social isolation or avoiding physical activity [7, 8]. Because of these detrimental physical and psychological impacts on older people, falling is a major public health problem [6]. However, it is also known that up to 40 % of falls are potentially preventable [9]. Assessment of frailty should therefore lead to interventions to reduce falls.

Multiple longitudinal studies have previously investigated frailty as a predictor of future falls in community-dwelling older populations [10–20]. Most of them demonstrated that frail older people were more likely to fall than were their non-frail counterparts, [10–17] but a few studies did not [18–20]. Their follow-up periods were one year [10–14] or longer, up to eight years [17] and it is not known if frailty is predictive of future fall risk over a short period of follow-up.

The objective of this study was to examine the associations between baseline frailty status and short-term incidence of future falls among community-dwelling older people who participated in an exercise promotion trial. We hypothesised frailty would predict future falling during the short follow-up period of 24 weeks.

## Methods

### Study population

The cohort of this study consisted of British community-dwelling older people in the usual care arm of a randomised controlled trial conducted in London and Nottingham/Derby in 2008–2013 to examine the effects of two exercise programs [21]. Included in this trial were people age > =65 years who were able to walk independently and participate in group exercise classes without unstable medical conditions. They were excluded if they had three or more falls in the previous year or were exercising for 150 min or more per week. Trial participants were more physically active than the older population, with lower levels of comorbidity and polypharmacy [21]. This trial was approved by the Nottingham Research Ethics Committee 2, National Health Service Nottinghamshire County and Westminster, Brent, Harrow, Hounslow and Barnet & Enfield Primary Care Trusts and was registered in ClinicalTrials.gov (NCT00726531) and ISRCTN (ISRCTN43453770). All participants provided written informed consent. Of a total of 1254 participants, 457 were allocated to the usual care arm. Those who did not return more than half of their falls diaries (*n* = 193) and those who had missing data on more than two variables out of 40 (5 %) for constructing the Frailty Index (FI) (*n* = 16) were excluded, leaving 248 participants for the analysis.

### Predictor variable: Frailty

Frailty was measured using FI constructed based on 40 deficits at baseline [22]. Of 248 participants included in this study, 222, 25 and 1 had 40, 39 and 38 variables available, respectively, to construct FI. The deficits are symptoms, signs, disabilities and diseases that are biologically sensible, accumulate with age, do not peak too early and cover a range of systems [23]. Although there is no standard set of deficits to construct FI, it is recommended to use at least 30–40 variables to accurately predict adverse outcomes and FIs based on different deficits appear to yield similar results [23, 24]. The deficits used in this study included 16 physical limitations, including activities of daily living and instrumental activities of daily living, 15 comorbidities, four psychological symptoms and one deficit each for obesity, polypharmacy, general health, low activity and pain (Table 1). Dichotomised deficits were scored as 1 if the deficit was present and as 0 if absent, while continuous or ordinal deficits were given a score between 0 and 1 to represent severity of the deficits. Some of the deficits were derived from the 12-item Short Form Survey and the ConfBal Scale, [25, 26] which were asked at the baseline of the trial [21]. FI was calculated by adding the scores of the deficits and dividing by the total number of the deficits available for each participant. In case of missing data, missing variables were excluded from both numerator and denominator. For example, if a participant had information of 38 deficit variables available (two missing) and had five deficits out of the 38, FI was calculated as 5 divided by 38 equals 0.13. FI can range from 0 (no deficit) to 1 (maximum deficits possible). Frailty was defined as FI > =0.25 according to previous studies [27, 28].

**Table 1:** List of 40 deficits for constructing frailty index

	Variable	Grading
	Physical/ADL/IADL limitations (*n* = 16)	
1	Difficulty with public transportation	Present = 1, absent = 0
2	Difficulty with moderate activity	Limited a lot = 1, limited a little = 0.5, not limited at all = 0
3	Difficulty with climbing stairs	Limited a lot = 1, limited a little = 0.5, not limited at all = 0
4	Difficulty with work activity	Limited a lot = 1, limited a little = 0.5, not limited at all = 0
5	Difficulty with sitting in chair	Not confident = 1, slightly confident = 0.5, confident = 0
6	Difficulty with getting up of chair	Not confident = 1, slightly confident = 0.5, confident = 0
7	Difficulty with picking up something	Not confident = 1, slightly confident = 0.5, confident = 0
8	Difficulty with standing unsupported	Not confident = 1, slightly confident = 0.5, confident = 0
9	Difficulty with walking indoors	Not confident = 1, slightly confident = 0.5, confident = 0
10	Difficulty with walking up slope	Not confident = 1, slightly confident = 0.5, confident = 0
11	Difficulty with walking down slope	Not confident = 1, slightly confident = 0.5, confident = 0
12	Difficulty with walking over uneven pavement	Not confident = 1, slightly confident = 0.5, confident = 0
13	Difficulty with walking down stairs indoors	Not confident = 1, slightly confident = 0.5, confident = 0
14	Difficulty with walking up stairs indoors	Not confident = 1, slightly confident = 0.5, confident = 0
15	Using walking aids	Yes = 1, no = 0
16	Balance problem	Present = 1, absent = 0
	Comorbidities (*n* = 15)	
17	Respiratory disease	Present = 1, absent = 0
18	Heart/circulatory disease	Present = 1, absent = 0
19	Endocine/metabolic disease	Present = 1, absent = 0
20	Musculoskeletal disease	Present = 1, absent = 0
21	Digestive disease	Present = 1, absent = 0
22	Nervous disease	Present = 1, absent = 0
23	Mental disease	Present = 1, absent = 0
24	Eye disease	Present = 1, absent = 0
25	Genitourinary disease	Present = 1, absent = 0
26	Neoplasms/benign growth disease	Present = 1, absent = 0
27	Infectious disease	Present = 1, absent = 0
28	Ear disease	Present = 1, absent = 0
29	Blood/related disease	Present = 1, absent = 0
30	Skin disease	Present = 1, absent = 0
31	other disease	Present = 1, absent = 0
	Psychological (*n* = 4)	
32	Feeling calm and peaceful	all of the time/most of the time = 0, some of the time = 0.5, a little of the time/none of the time = 1
33	Having a lot of energy	all of the time/most of the time = 0, some of the time = 0.5, a little of the time/none of the time = 1
34	Feeling downhearted and low	all of the time/most of the time = 1, some of the time = 0.5, a little of the time/none of the time = 0
35	Social activity interfered by physical health or emotional problems	all of the time/most of the time = 1, some of the time = 0.5, a little of the time/none of the time = 0
	Others (*n* = 5)	
36	Obesity	BMI ≥30 = 1, BMI <30 = 0
37	Polypharmacy	≥6 medications = 1, <6 medications = 0,
38	Self-rated general health	Poor/fair = 1, good = 0.5, very good/excellent = 0
39	Low activity	no exercise = 1, exercise once in a while = 0.5, regular exercise = 0
40	Normal work interfered by Pain	Extremely/quite a bit = 1, moderately = 0.5, a little bit/not at all = 0

### Outcome variable: Future falls

For the purposes of this study, a fall was defined as an event of unintentionally coming to rest on the ground, floor, or other lower level [5]. Participants who had at least one fall during the 24-week follow-up period were defined as fallers and participants who did not have falls were defined as non-fallers. Falls were monitored prospectively over the study period using falls diaries. The falls diaries were mailed to each participant every four weeks, for a total of six diaries, and participants were required to record daily if they had fallen or not [29]. All participants who did not return the diary were reminded by a phone call.

### Other covariates

Socio-demographic and clinical information collected at baseline included age, gender, height, weight, ethnicity, highest level of education achieved, annual household income and number of falls in the previous year. Height and weight were measured at the baseline examination and body mass index (BMI) was calculated as weight in kilograms divided by square of height in meters.

### Statistical analyses

Baseline socio-demographic characteristics were compared using Mann–Whitney *U* test for continuous variables and chi-square tests for categorical variables between fallers and non-fallers. The Spearman’s correlation coefficient was calculated to assess correlations between FI and the socio-demographic characteristics. Odds ratios (OR) with 95 % confidence intervals (95 % CI) of frailty (dichotomous, FI > =0.25) and FI (continuous, per 0.01 increment) for future fall risks were calculated using logistic regression models, unadjusted and adjusted for age, gender and variables significantly associated with future falls in the univariate models. We used a receiver operating characteristic (ROC) curve analysis to assess FI’s ability to predict future falls and calculated the area under the ROC curve (AUC). All statistical analyses were two-sided, with an alpha level of 0.05 and were performed with IBM SPSS Statistics (version 20, IBM Corporation, Armonk, NY, USA).

## Results

Applying the FI to the study cohort identified 45 frail and 203 non-frail participants (Table 2). Frail participants were older, had a higher BMI, were more likely to live in Nottingham and have reported more falls in the previous year than non-frail participants.

**Table 2:** Baseline characteristics for study cohort, by frailty status

Variable^a^	Entire cohort	Frail^b^	Non-frail^b^	p value
	*N* = 248	*n* = 46	*n* = 202	
Age	72.9 ± 6.1	75.9 ± 7.2	72.2 ± 5.6	0.001
Female	158 (63.7 %)	31 (67.4 %)	127 (62.9 %)	0.57
Body mass index	26.4 ± 4.9	29.2 ± 6.6	25.8 ± 4.2	<0.001
White ethnicity	223 (90.7 %)	43 (93.5 %)	180 (90.0 %)	0.47
Education				
College/University	124 (50.4 %)	21 (45.7 %)	103 (51.5 %)	0.47
Primary/Secondary	122 (49.6 %)	25 (54.3 %)	97 (48.5 %)	
Income				
up to £20000	135 (61.4 %)	23 (59.0 %)	112 (61.9 %)	0.74
£20001+	85 (38.6 %)	16 (41.0 %)	69 (38.1 %)	
Site				
London	99 (39.9 %)	12 (26.1 %)	87 (43.1 %)	0.03
Nottingham	149 (60.1 %)	34 (73.9 %)	115 (56.9 %)	
Number of falls in the previous year	0.30 ± 0.56	0.48 ± 0.69	0.26 ± 0.52	0.05
0	187 (75.4 %)	29 (63.0 %)	158 (78.2 %)	0.05
1	48 (19.4 %)	12 (26.1 %)	36 (17.8 %)	
2	13 (5.2 %)	5 (10.9 %)	8 (4.0 %)	

^a^mean ± standard deviation or n (%)

^b^Frail was defined as frailty index > =0.25 and non-frail was defined as frailty index <0.25

Table 3 presents baseline characteristics of the study cohort and compares fallers and non-fallers. The mean age was 72.9 years old and 63.7 % were women. Of 248 participants, 46 (18.5 %) were classified as frail at baseline and 57 (23.0 %) had at least one fall during the 24-week study period. The mean FI was 0.16 (standard deviation 0.11) in the entire cohort. Fallers had higher mean FI (0.21) compared to non-fallers (0.14) and more fallers were classified as frail (19/57, 33.3 %) compared to non-fallers (27/191, 14.1 %). Given those who had had three or more falls in the previous 12 months were excluded at the time of the trial enrollment, all participants had had no or up to two falls in the previous 12 months. As expected, because a previous history of falls is a strong predictor of future falls, fallers were more likely to have fallen twice over the previous year (8/57, 14.0 %) than were non-fallers (5/191, 2.6 %). There were no significant differences between fallers and non-fallers in age, gender, BMI, ethnicity, education, income, enrollment site and the mean number of falls in the previous year.

**Table 3:** Baseline characteristics for study cohort, by fall status

Variable^a^	Entire cohort	Fallers^b^	Non-fallers^b^	p value
	*N* = 248	*n* = 57	*n* = 191	
Age	72.9 ± 6.1	72.9 ± 6.3	72.9 ± 6.1	0.94
Female	158 (63.7 %)	38 (66.7 %)	120 (62.8 %)	0.60
Body mass index	26.4 ± 4.9	26.8 ± 3.5	26.3 ± 5.3	0.14
White ethnicity	223 (90.7 %)	55 (96.5 %)	168 (88.9 %)	0.08
Education				
College/University	124 (50.4 %)	30 (52.6 %)	94 (49.7 %)	0.70
Primary/Secondary	122 (49.6 %)	27 (47.4 %)	95 (50.3 %)	
Income				
up to £20000	135 (61.4 %)	27 (51.9 %)	108 (64.3 %)	0.11
£20001+	85 (38.6 %)	25 (48.1 %)	60 (35.7 %)	
Site				
London	99 (39.9 %)	21 (36.8 %)	78 (40.8 %)	0.59
Nottingham	149 (60.1 %)	36 (63.2 %)	113 (59.2 %)	
Number of falls in the previous year	0.30 ± 0.56	0.40 + 0.73	0.27 + 0.50	0.19
0	187 (75.4 %)	42 (73.7 %)	145 (75.9 %)	0.002
1	48 (19.4 %)	7 (12.3 %)	41 (21.5 %)	
2	13 (5.2 %)	8 (14.0 %)	5 (2.6 %)	
Frailty (Frailty Index > =0.25)	46(18.5 %)	19 (33.3 %)	27 (14.1 %)	0.001
Frailty Index	0.16 ± 0.11	0.21 ± 0.15	0.14 ± 0.10	0.006

^a^mean ± standard deviation or n (%)

^b^Fallers were defined as those who had one or more falls and non-fallers were those who did not have any falls during the study period

Tables 4 and 5 show univariate and multivariate logistic regression models, respectively. Both frailty and FI at baseline were significantly associated with future falls during the follow-up period in univariate logistic regression models; each 0.01 increase in FI was associated with 5 % increased odds of future falls (OR = 1.05, 95 % CI = 1.02–1.07, *p* < 0.001) and those with frailty were approximately three times more likely to fall during the follow-up period than were those without (OR = 3.04, 95 % CI = 1.53–6.02, *p* = 0.001). In multivariate logistic regression models, the associations between frailty status and future falls persisted after adjusting for age, gender and history of two falls in the previous year (OR = 1.05, 95 % CI = 1.02–1.08, *p* = 0.001; OR = 2.95, 95 % CI = 1.41–6.17, *p* = 0.004, respectively).

**Table 4:** Univariate logistic regression models predicting falls during the 24-week follow-up

Variable	Odds Ratio (95 % CI)	p value
Frailty (Frailty Index > =0.25)	3.04 (1.53–6.02)	0.001
Frailty Index (per 0.01 increase)	1.05 (1.02–1.07)	<0.001
Age	1.00 (0.96–1.05)	0.93
Female gender	1.18 (0.63–2.21)	0.60
White ethnicity	0.29 (0.07–1.28)	0.10
Education (college/university)	1.12 (0.62–2.03)	0.70
Income (£20001+)	1.67 (0.89–3.13)	0.11
Site (London)	0.85 (0.46–1.56)	0.59
Number of falls in the past year	1.49 (0.91–2.42)	0.11
Any falls in the past year	1.13 (0.57–2.21)	0.73
Two falls in the past year	6.07 (1.90–19.39)	0.002

**Table 5:** Multivariate logistic regression models predicting falls during the 24-week follow-up

	Model 1^a^		Model 2^b^	
Variable	Odds Ratio (95 % CI)	p value	Odds Ratio (95 % CI)	p value
Frailty (Frailty Index > =0.25)	2.95 (1.41–6.17)	0.004	-	-
Frailty Index (per 0.01 increase)	-	-	1.05 (1.02–1.08)	0.001
Age	0.99 (0.94–1.04)	0.66	0.98 (0.92–1.03)	0.37
Female gender	1.12 (0.58–2.14)	0.74	1.15 (0.59–2.21)	0.69
Two falls in the past year	5.17 (1.56–17.10)	0.007	5.06 (1.47–17.39)	0.01

^a^Model 1 used frailty (Frailty Index > =0.25) as an independent variable adjusted for age, gender, and history of two falls in the past year

^b^Model 2 used Frailty Index as an independent variable adjusted for age, gender, and history of two falls in the past year

**Fig. 1:**
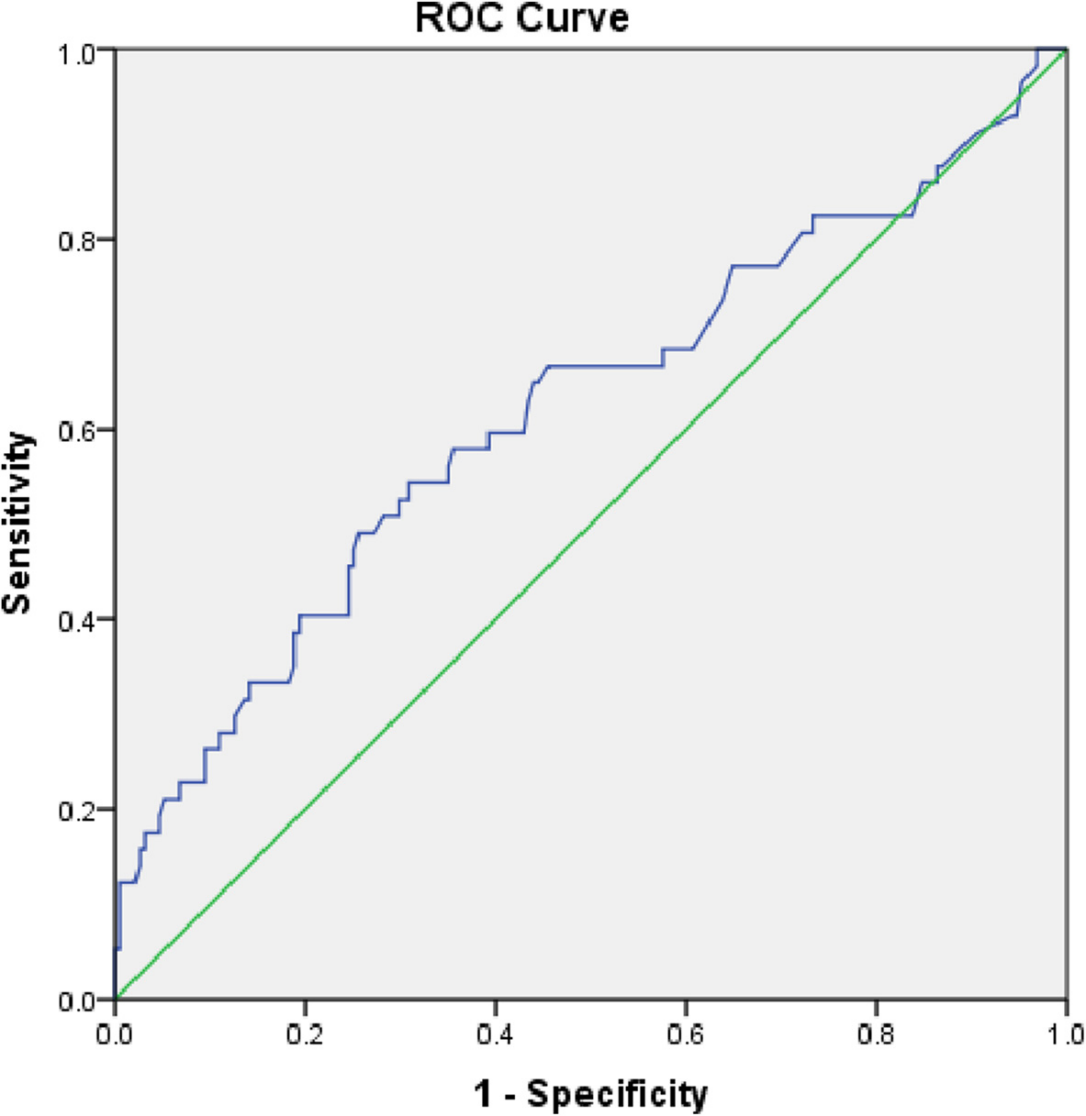
Receiver operating characteristic curve using frailty index as a predictor of future falls. ROC: Receiver operating characteristic. Area under the receiver operating characteristic curve = 0.62, 95 % confidence interval 0.53–0.71, *p* < 0.01

Figure 1 displays a ROC curve of FI as a predictor of future falls. In this population sample FI predicted future falls with fair accuracy with AUC of 0.62 (95 % CI = 0.53–0.71, *p* = 0.006). Sensitivity, specificity, positive predictive value, negative predictive value, positive likelihood ratio and negative likelihood ratio of using a FI of 0.25 as a cut-off point to define frailty, were 31.6 %, 85.9 %, 40.0 %, 80.8 %, 2.23 and 0.80, respectively.

## Discussion

This study of 248 British community-dwelling older people demonstrated that frailty was a significant predictor of future falls during the short follow-up period of 24 weeks independent of history of falls, which is a strong predictor of falls risk, and other covariates.

In the literature, two previous studies using FI demonstrated significant associations between frailty and future falls after longer follow-up periods than this study [14, 17]. FI based on 34 deficits at baseline predicted incident falls during the third year in approximately 4000 women aged 55 years or older in Canada (OR = 1.02 for 0.01 increase in FI, 95 % = 1.02–1.03) [14]. Another study constructed FI using 33 deficits and divided a cohort of 3257 community-dwelling Chinese people from the Beijing Longitudinal Study of Aging study into five subgroups based on FI (<=0.03, 0.03–0.10, 0.10–0.20, 0.21–0.5 and >0.5). They showed that higher FI was associated with higher risk of recurrent falls over the subsequent eight years (OR = 1.54 for each FI subgroup increment, 95 % = 1.34–1.76) [17]. Unlike our study, these studies did not use a specific cut-off point to define frailty.

Frailty defined by other definitions, including a Fried’s phenotype,[11, 12, 15, 16] Study of Osteoporotic Fractures (SOF) index,[11, 12, 15] Longitudinal Aging Study Amsterdam frailty instrument,[10] Canadian Study of Health and Aging Clinical Frailty Scale,[13] and Conselice Study of Brain Aging index [20] have also been shown to predict future falls in community-dwelling populations. Of these, two studies performed ROC curve analysis and showed AUC, based on Fried’s phenotype and SOF index, ranging from 0.61 to 0.63, which was comparable with our result using FI (AUC = 0.62) [11, 12].

The findings of this study need to be interpreted cautiously. This study was performed by a secondary analysis using data from the cohort originally recruited for an exercise intervention trial. The participants, who volunteered for the exercise intervention trial, may have been a selected group of people who were more motivated to exercise and adopted a healthy lifestyle. Therefore, our findings may not be completely generalisable. This is perhaps seen in the comparison of the cohort based on the FI (Table 2), where we may have expected female gender, lower income and lower education to have been significantly different between those identified as frail and those as non-frail, based on previous literature. However, these associations were not seen. We excluded frequent fallers, those who had three or more falls in the previous year, at the baseline, which may have led to underestimating the association between frailty and future fall risk. The most frequently used frailty definition, Fried’s phenotype, was not used to define frailty in this study due to the unavailability of necessary data. However, although it may be time-consuming to use FI in clinical practice as many of the deficit variables are not routinely collected, FI is another popular definition that has been examined and validated in various populations and settings and has been shown to predict mortality more accurately than does the Fried’s phenotype model [27]. A significant proportion, approximately 40 % (193/457), of participants allocated to the usual care arm returned between no and three falls diaries out of six and were therefore excluded. Compared with those who returned four to six diaries, the excluded participants were significantly frailer (mean FI 0.22 vs. 0.16, *p* < 0.001), which may have underestimated the fall risk. These two groups did not differ in mean age, gender, or history of two falls in the previous year. Lastly, we did not have data on some important potential confounders, such as cognitive function, alcohol use or high risk medication use, and could not control for these factors.

The major strength of this study is high quality incident fall data based on the comprehensive fall monitoring system. The standardised protocol included participant’s daily recordings in the falls diaries and the submission of the diaries at short intervals, along with follow-up reminder phone calls if necessary. Furthermore, only participants who returned more than half of the fall diaries were included in the analyses. These procedures may have contributed to minimising recall bias and avoiding underreporting falls [30].

Another strength are the clinical implications of our findings. This study cohort was derived from a large exercise intervention trial, where the participants were people aged over 65 years who were recruited in primary care and volunteered for the trial. Those at high risk for falling (i.e. with history of three or more falls in the previous year, unstable medical conditions, or mobility disability) were excluded. These relatively well older people without high risks for falling can be seen as “ageing well”. Nevertheless, among older people who are ageing well, frailty (defined as FI > =0.25) identified those with higher risk of falling in the next six months. This information may allow primary care physicians and geriatricians to intervene earlier in the falls trajectory, and researchers to design more effective exercise interventions or fall prevention programs [31].

## Conclusions

In summary, frailty based on the deficit accumulation model of FI was a significant predictor of the short-term incidence of future falls among British community-dwelling older people. Given the fact falling can have negative impacts on older people, it is important for healthcare practitioners working with older people to recognise frailty as a risk factor for imminent falling, even in those who appear to be ageing well.

## Abbreviations

95%CI: 95 % confidence interval
AUC: Area under the receiver operating characteristic curve
BMI: Body mass index
FI: Frailty index
OR: Odds ratio
ROC: Receiver operating characteristic
SOF: Study of Osteoporotic Fractures
